# Computational Psychiatry of ADHD: Neural Gain Impairments across Marrian Levels of Analysis

**DOI:** 10.1016/j.tins.2015.12.009

**Published:** 2016-02

**Authors:** Tobias U. Hauser, Vincenzo G. Fiore, Michael Moutoussis, Raymond J. Dolan

**Affiliations:** 1 Wellcome Trust Centre for Neuroimaging, University College London, London, WC1N 3BG, UK; 2 Max Planck UCL Centre for Computational Psychiatry and Ageing Research, London, WC1B 5EH, UK

**Keywords:** Attention-deficit hyperactivity disorder (ADHD), neural gain, dopamine, noradrenaline, norepinephrine, computational psychiatry, behavioural variability

## Abstract

Attention-deficit hyperactivity disorder (ADHD), one of the most common psychiatric disorders, is characterised by unstable response patterns across multiple cognitive domains. However, the neural mechanisms that explain these characteristic features remain unclear. Using a computational multilevel approach, we propose that ADHD is caused by impaired gain modulation in systems that generate this phenotypic increased behavioural variability. Using Marr's three levels of analysis as a heuristic framework, we focus on this variable behaviour, detail how it can be explained algorithmically, and how it might be implemented at a neural level through catecholamine influences on corticostriatal loops. This computational, multilevel, approach to ADHD provides a framework for bridging gaps between descriptions of neuronal activity and behaviour, and provides testable predictions about impaired mechanisms.

## Trends

ADHD is one of the most common psychiatric disorders during childhood, but the neurocognitive mechanisms behind it remain elusive.

Behaviourally, ADHD is best characterized by increased variability across multiple cognitive domains and timescales.

By using Marr's three levels of analysis, we show how impairments in neural gain can explain ADHD abnormalities, spanning from behaviour to neural activity.

On an algorithmic and implementation level, we show how increased variability can be caused by neural gain impairments, and how it can be modelled using reinforcement learning and corticostriatal network models.

We furthermore show how these levels can be linked to impairments in catecholamine systems (dopamine and noradrenaline).

## The Need for a Better Neurocomputational Understanding of ADHD

Maintaining one's mental focus is hard, especially when reading a dry and complicated paper. Suddenly you would rather clean the kitchen or surf the Internet. Nevertheless, most people maintain focus and persist with the task at hand. Neurobiologically, we propose that the **catecholaminergic brain systems** (see Glossary) modulate attention [1] by increasing the neural gain and, thus, suppressing cognitive switching [2] (Box 1).

For 5% of the population, the ability to focus is disturbed to an extent that strongly affects their daily functioning. Many are diagnosed with **ADHD** [3], a developmental psychiatric disorder thought to arise, in part, out of a genetic vulnerability [4]. ADHD is characterised by inattention, hyperactivity, and/or impulsivity [5] and its negative effects on a person's occupational success, wellbeing, and health risks (e.g., for substance abuse [6]) make it important to understand this disorder.

Research on ADHD has intensified since the early 1990s [6] without clear candidate genes or brain response patterns predicting the disorder having been identified. There is no unifying theory explaining the pathophysiology of ADHD. Indeed, current classification criteria are likely to subsume multiple brain disorders with a similar behavioural expression within the label ‘ADHD’.

Here, we use a multilevel approach to propose that ADHD crucially involves an impairment of neural gain modulation leading to inappropriately variable behaviour. By using **Marr's three levels of analysis** [7] (Box 2), we show how it is possible to translate behavioural findings into mathematical algorithms and neural circuit impairments (and vice versa). This approach also provides fruitful hypotheses about potential neurobiological subgroups, which could be the object of future investigation.

## Neurocognitive Impairments in ADHD

To understand a psychiatric disorder, it is important to unite several levels of impairments spanning symptoms, behaviour, neural, and neurochemical markers. Here, we selectively review the most consistent neurocognitive impairments and go on to argue that these can all be explained by impaired neural gain.

### Behavioural Markers: The Consistency of Inconsistencies

Behavioural findings in ADHD are numerous, and here we confine ourselves to a general pattern of ADHD-related impairments consistently present across domains and tasks.

#### Reaction Time Variability

One of the most consistent findings in subjects with ADHD is an increase in reaction time (RT) variability (such as RT standard deviations). This is reliably found across many tasks, laboratories, and countries [8] and is one of the best behavioural classifiers for ADHD [9].

#### Response Inconsistencies

Simple response tasks, such as the **continuous performance task** (CPT, Box 3), require a participant to respond to prelearned target stimuli while withholding an action for nontarget stimuli. This simple response-to-target, nonresponse-to-nontarget pattern is used in a variety of task settings that investigate different cognitive domains, such as attention (alertness, vigilance, and sustained attention tasks), response inhibition (Go/NoGo and Flanker tasks), or working memory (n-back tasks). Across all these tasks, patients with ADHD generally make less target-related responses (**errors of omission**) and more nontarget responses (**errors of commission**) [10]. Subsequently, we use the CPT as an example of these response biases and to illustrate how these impairments can be caused by decreased neural gain (Box 3, Box 4).

#### Decision Making and Reward Learning

In the context of neuroeconomic approaches to behaviour, decision-making has received considerable attention from the ADHD community 11, 12, 13, 14, 15. However, relatively few studies have used neuroeconomic tasks and models that address actual mechanisms and their putative impairment in ADHD. In one of the first such studies, Hauser *et al.* [16] investigated decision-making in adolescent patients with ADHD using learning models and found that an increased **decision temperature** parameter (Box 3) accounted for the more stochastic behaviour seen in ADHD. This is in line with previous computational and animal work relating ADHD-like behaviours to decision temperature [17]. Other studies investigated **delay gratification** and **temporal discounting** to study impulsivity in ADHD. While such initial reports suggested increased discounting in ADHD, more recent studies reveal a more complex picture [18]. However, we note evidence that increased discounting is strongly associated with increased choice variability [19].

### Neural Markers: The Catecholaminergic Systems

In contrast to other psychiatric disorders, ADHD has relatively few candidate neurotransmitter systems. Studies from different fields have converged on the catecholamine neurotransmitter systems (Box 1) dopamine (DA) and noradrenaline (NA) as contributing to the impairments seen in ADHD 13, 20, 21, 22, 23.

Methylphenidate is a highly effective treatment in ADHD whose mode of action is a targeting of dopaminergic reuptake from synaptic cleft [24]. By preferentially blocking the re-uptake of DA, methylphenidate increases synaptic DA and, hence, dopaminergic transmission. Nonstimulant medications, such as atomoxetine, more specifically target the noradrenergic system in prefrontal areas and may be more effective in patients with a putative deficit in NA regulation [25]. While atomoxetine prevents NA from being removed from the synaptic cleft, other drugs specifically stimulate α2-adrenoceptors rather than acting on all NA receptor types 22, 26.

A source of more direct evidence comes from human **positron emission tomography** (PET) and animal studies that suggest a hypofunction in a DA system in striatal and prefrontal areas in ADHD 13, 14, 21, 23, 27. Less evidence is available for NA involvement due to methodological reasons [21]. In addition, genetic studies implicate DA- and NA-related genes in ADHD 6, 22, 28.

In line with the relatively widespread effects of neural gain in the brain [29], functional neuroimaging in ADHD has revealed multiple brain networks as affected 6, 30, including the striatum [15] and medial prefrontal cortex 16, 30. It is of interest that both are densely innervated and modulated by catecholamines 31, 32, 33 and show deficient functioning during task performance and at rest 30, 34.

## Deficient Neural Gain Modulation in ADHD

Here, we illustrate how lowering neural gain at the neurophysiological (implementation) and algorithmic levels can induce ADHD-like neurocognitive impairments. To understand why the brain uses neural gain modulation to guide behaviour in the first place, we first discuss the importance of balancing between choice stability and choice variability from a theoretical standpoint.

### Computational Level: Why Arbitrate between Stable and Unstable Behaviours?

So far, we have concluded that a consistent feature of ADHD is an increased variability in behaviour. According to Marr, the first level of analysis should describe the problem a system (i.e., the brain) faces and how it tries to solve it [7]. So why should the healthy brain allow for substantial behavioural variability? Why does the brain not always select the option with the highest returns according to the information available? Why do we sometimes go for options that are not the best and explore? We note that this is not about simple imperfection, because there are numerous biological functions that are executed with engineering precision.

The dilemma that the brain has to solve arises from acting in environments where different options may change their value for the subject. Agents not only have to exploit the option it estimates as the best, but must also explore the value of alternative options so as to gather more information 35, 36. One example is foraging, where different trees may change the amount of fruits they carry. Thus, it is more adaptive to occasionally try alternative trees. This might be particularly important in a developmental context, where a child has a limited prior knowledge about an environment and, thus, can profit from exploring unknown environments.

From both a reinforcement learning and information theoretic perspective, the arbitration between different options is construed as balancing ‘exploitation’ and ‘exploration, information gathering’. This is a hard problem to solve, but there are simple, well-established, methods, such as randomly sampling from one's beliefs, or Thomson sampling [37]. Recent neuroscientific work suggests that both immediate utility and information gathering drive our behaviour [38]. We note that controlled addition of noise to a system to optimise its behaviour is by no means confined to decision-making and applies to many problem-solving systems (e.g., stochastic resonance or simulated annealing).

The increased variability in ADHD can be seen as altered exploitation–exploration trade-off. In paradigms with no uncertainty, increased exploration makes no sense; by contrast, in a natural environment, the optimal amount of attentional stability, in view of uncertainty, is a matter of degree. Moving to a societal level, increased exploratory behaviour in a proportion of the population may be advantageous. Simulations by Williams and Taylor [39] demonstrate that groups with 5% of ADHD-like agents show optimal foraging behaviours and increased survival, and may explain why ADHD remains prevalent in the population despite its negative effects on the individual.

In summary, the brain has to arbitrate between either exploiting currently preferred options or sampling alternatives and learn from experience. While low exploration in most members of a group ensures stability, a low proportion of people with ADHD allows learning from exploration and, thus, can be evolutionarily beneficial for a group.

### Algorithmic Level: How to Arbitrate between Exploitation and Exploration?

The second level of Marr asks how a problem is solved. Specifically, it asks for mathematical descriptions of how the system solves its task. In recent years, these approaches have gained increased interest. Bayesian reasoning and reinforcement learning theories in particular have provided biologically useful algorithms that the brain appears to exploit 40, 41, 42, 43, 44.

Can reinforcement learning account for behavioural variability across different tasks and cognitive domains? In Box 3, we propose that increased variability can be explained by an altered action selection process. At the core of this action, selection process is the **decision temperature parameter** *τ*, a measure of choice stochasticity. It describes to what extent the agent sticks to what it effectively believes to be the best choice. Higher decision temperatures make the agent more likely to choose from options currently estimated to have less-than-maximum values. By contrast, lower temperatures make the agent choose the highest value option more often, thereby avoiding alternatives even if they have almost the same value (Box 3). Thus, increasing *τ* elicits more variable behaviours, even in simple stimulus–response tasks. A similar effect has been shown in the context of delay gratification [17]. It is important to note that, in temporal discounting, subjects with high temperatures also tend to have high discounting preferences [19]. Lower temperatures are good for exploiting current beliefs, while higher ones help exploration of uncertain options, as well as evening out resource utilisation.

In the context of learning and decision-making, previous theories 12, 14 proposed that impaired learning would elicit ADHD-like behaviour, driven by impoverished **reward prediction error** (RPE) signals. However, recent empirical data that addressed learning and decision-making in ADHD demonstrated that ADHD participants are not well characterized by impaired learning, but instead by an increased decision temperature [16].

We can tentatively conclude that increased variability at the algorithmic level is explained by an increased decision temperature in relation to an action selection process. We suggest that this is likely to be underpinned by lowered neural gain, potentially caused by malfunctioning catecholamine systems (Box 3) and altered connectivity 45, 46. Neural underpinnings apart, an understanding of the key deficits of ADHD at the algorithmic (information-processing) level may inform learning-based treatments for this disorder, for which there is currently great demand but limited evidence as to their efficacy 47, 48.

### Implementation Level: How Does Gain Affect Computations in Neural Loops?

The implementational level asks how the algorithm functions of the second level are realised in neural hardware, that is, in this instance, how the brain circuits instantiate and dynamically select between different options, what structural change is associated with an hypothesised neural gain impairment, and how this is translated into behavioural dysfunctions.

Neural models of corticostriatal circuits provide tools to study a catecholaminergic modulation of behavioural selection processes, such as the effects of reduced DA in striatal areas. Since refined maps of DA receptor distributions in the striatum are established, the majority of these models investigate the role of DA 49, 50. Such models describe how information is propagated from the striatum to the cortex (and back) through multiple pathways, and how these loops process and represent complex information. Striatal DA has a crucial role in this information processing (Box 4) and these models have been successful in describing neural processes underlying motor impairments in disorders such as Parkinson's disease (PD) 51, 52, 53. Previous corticostriatal models have also been successful in describing ADHD-like response inhibition and working memory deficits, but do not explain increased response variability through DA impairments [23]. Recent refinements in understanding the specific functions of the basal ganglia pathways 54, 55 have led to a substantial change in how we think of a D2-driven indirect pathway that allows us to account for ADHD-related variability by means of DA impairments (Box 4) 56, 57. This has also facilitated an understanding of why the same pharmacological increase in DA can improve disorders that are at the opposite side of a motor activity spectrum, namely ADHD and PD. Few frontostriatal loop models have considered the contribution of other catecholamines, such as NA. Notably, Frank *et al.* [23] showed that impaired NA function increased behavioural variability as seen in ADHD by changing neural gain in prefrontal areas.

The aforementioned models of corticostriatal loops demonstrate that multiple impairments in neural gain (such as decreased frontal NA [23] or lowered striatal DA efficacy [56]) can cause increased behavioural variability. This raises interesting new questions that can be addressed in future behavioural, modelling, and (pharmaco-) neuroimaging work. Key here is to understand how different catecholamines can be dissociated, not only in terms of their impact on behaviour, but also with respect to the neural correlates of these impairments. Moreover, it is important to determine which receptor types are involved in ADHD. We consider it likely that different ADHD subgroups can be characterised by specific receptor impairments and, thus, a specific neurocognitive pattern. For example, our corticostriatal loop models 2, 56 suggest that neural gain impairments can be caused by either reduced DA release in the striatum or impairment at the level of D1 or D2 receptors. Current PET studies support an impaired striatal DA release as well as changes in D2 receptor density [21]. For NA, ADHD has mainly been associated with impairments in α2-adrenoceptors 26, 58, known to boost prefrontal representations 58, 59. More recent evidence also highlights the importance of β-adrenoceptors for modulating neural gain [60]. Only by finding specific neurocognitive markers of catecholaminergic impairment, we will be able to obtain neurobiologically valid ADHD subtypes and, thus, refine the targeting of pharmacological therapy (see Outstanding Questions). Moreover, such refinements of ADHD subtypes could facilitate nonpharmacological interventions, such as neurofeedback 47, 61, 62, 63, 64 and transcranial brain stimulation, allowing a focus on more specific neural substrates (Box 2).

## Concluding Remarks

To understand psychiatric disorders such as ADHD it is important to determine which neurocognitive processes go awry, and how. Psychiatry has traditionally suffered an explanatory gap between neurobiological mechanisms and symptom-level behaviours. Mathematical attempts to bridge different levels of description are few, but only by working across levels that span computational theory to neural implementation and back, can we better understand the neurocognitive impairments causing psychiatric disorders.

Here, we illustrate that ADHD can be described in terms of impaired neural gain across different levels of analysis. Based on the premise that the brain needs to arbitrate between exploration and exploitation, we show that an increased behavioural variability in ADHD can be expressed as neural gain impairments by an increased decision temperature parameter at an algorithmic level, as well as by catecholaminergic impairments at a neural implementation level.

Similarly, we can conceptualise key symptoms of ADHD as stemming from neural gain impairments. For example, inattention can be seen as a frequent shifting between different goals and an inability to stay with, and focus on, the currently most valuable option (as illustrated in Box 4). Likewise, decreased neural gain and, hence, behavioural switching may contribute to hyperactivity. By contrast, it can be conceptualised as akin to inattention, where frequent switches between cognitive goals propagate through the motor system and lead to frequent changes in motor programs, possibly characterising a combined ADHD subtype. A characteristic of such an impairment might be sudden standing up during class or the abrupt stopping of an ongoing behaviour. Alternatively, the neural gain impairments could only arise at a motor level, where one would expect markedly increased, undifferentiated motor actions and an inability to suppress evanescent, but inappropriate, motor response tendencies without marked inattentive symptoms (i.e., hyperactive-impulsive subtype).

Despite a likely heterogeneity in ADHD, we propose that neural gain modulation is a consistent impairment across many clinical subgroups. We can now hypothesise that ADHD subgroups may be better delineated by the specific profile of their neural gain impairments. One subgroup might primarily suffer from striatal DA impairment, expressing itself by more reward-related stochasticity and possibly striatal RPE impairments. Another subgroup might lack in frontal NA functioning, which might be expressed by impaired prefrontal signals and altered multiattribute processing. However, to be able to dissociate such subgroups, we need to develop better behavioural tasks and models, further advance computational neuroimaging, and develop neural models that are capable of dissociating different aspects of neural gain (see Outstanding Questions).

Outstanding Questions Can we dissociate different forms of neural gain impairment (e.g., DA versus NA; prefrontal versus striatal; α- versus β-adrenoceptor subtypes) behaviourally?

What are the unique features of NA and DA gain impairments behaviourally, algorithmically, and in neural loop models?

How can (computational) non-invasive neuroimaging contribute to dissociating different forms of neural gain impairment?

Can a neural gain-based classification of subgroups be predictive of pharmacological treatment efficiency? Can the understanding of the associated information processing inform psychotherapy?

## Figures and Tables

**Figure I:**
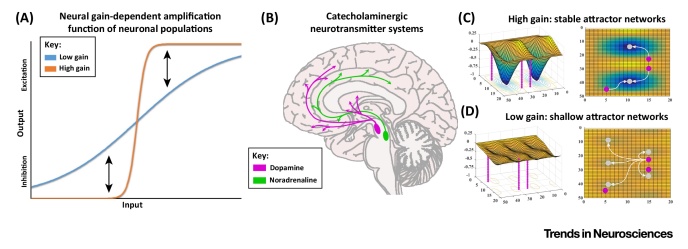
Neural Gain and Catecholamines. (A) Neural gain has an amplifying effect on neuronal signals by boosting strong inputs. (B) Catecholamine systems are crucial for modulating brain-wide neural gain. On a network-level, (C) high gain leads to stable attractor states and thus consistent outputs and behaviours, whereas (D) low gain causes unstable and shallow attractor states.

**Figure I:**
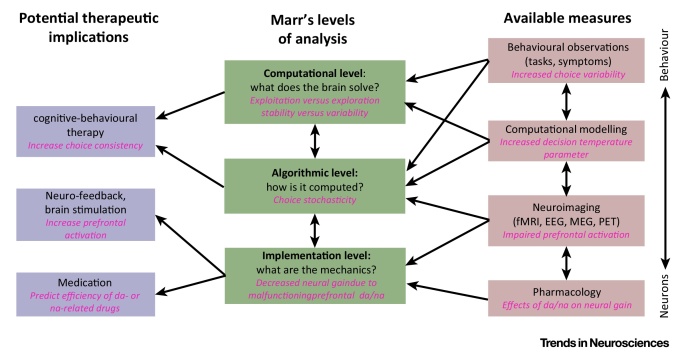
Modelling Psychiatric Disorders Across Marrian Levels of Analysis Helps Refining and Understanding the Mechanisms of these Disorders.

**Figure I:**
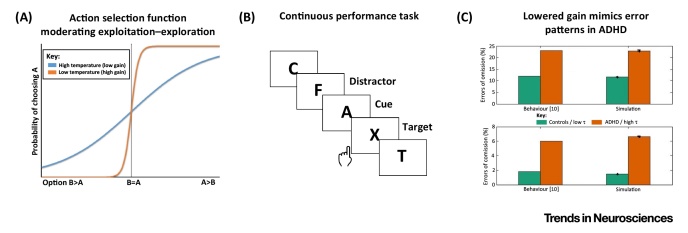
Algorithmic Level of Neural Gain Impairment. On the algorithmic level, (A) neural gain can be described by a change in the softmax decision steepness parameter. (B) Simulated data of the continuous performance task illustrates the effect of that parameter: (C) low gain renders behaviour more variable and ADHD-like (reference data from Losier et al. [10]).

**Figure I:**
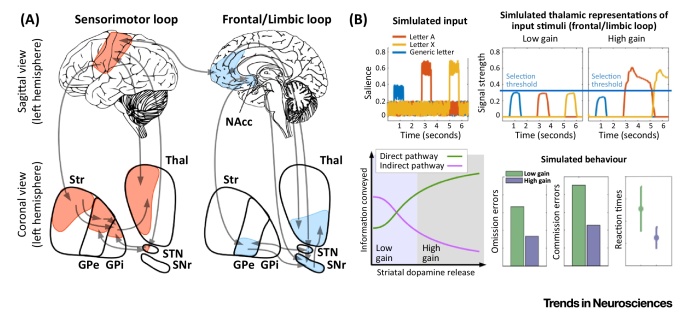
Neural Gain Impairments Drive Behavioral Variability in Corticostriatal Loops. (A) Corticostriatal loop models describe how information is processed and represented in these loops. (B) Under low neural gain, differentiation of representations is poor and behavior unstable. High gain leads to clearly differentiated representations and stable behavior. Abbreviations: GPe, globus pallidus externus; GPi, globus pallidus internus; NAcc, nucleus accumbens; SNr, substantia nigra; STN, subthalamic nucleus; Str, striatum; Thal, thalamus.

## Box 1 Neural Gain: Catecholamines Regulating Stability of Neuronal Systems

The brain can be thought of as a signal-processing machine that selects relevant information to act. Overburdening with information means that it needs to decide which aspects of its inputs to treat as important by boosting these relevant signals, and which aspects to treat as unimportant and attenuate. The brain cannot just rely on amplifying the strongest signal and filtering out everything else, but must keep a balance between competing signals according to environmental and internal demands. The degree to which neural signals are amplified or suppressed has been termed ‘neural gain’ and this effect can be mimicked by a sigmoidal function (Equation I):

[I] $$f_{G}\left(x\right)=\frac{1}{1+e^{-\left(Gx+B\right)}}\text{,}$$

where an input signal *x* is amplified by the neural gain factor *G* [70] (Figure IA).

In high neural gain states (Figure IA, orange), neural populations strengthen strong and attenuate weak incoming signals. This leads to neural representations that are less susceptible to noise [71]. Such states are most beneficial in conditions where the brain needs to avoid distraction, such as fleeing from a predator.

By contrast, in low neural gain states (Figure IB, blue), the system is not dominated by the most prevalent signals and, thus, it is more likely to detect weaker signals that may carry important information [29]. Such states can be helpful because weak, but important, information might be carried in a nondominant channel. For example, seeing the silhouette of a predator in the grass or in the periphery of vision.

Neural gain can also be related to **neural attractor** states: high gain leads to stable behaviours and attractor states where neural networks quickly converge to stable firing patterns (Figure IC, pink starting states quickly and consistently result in the same end states; *cf* supplemental information online). However, low gain is characterised by variable attractor states and behaviours (Figure ID, pink starting states end up in multiple unstable states).

Neural gain should affect widespread neural populations. Thus, it is not surprising that the catecholaminergic neurotransmitter systems [i.e., dopamine (DA) and noradrenaline (NA)] have been found to function as neural gain modulators 31, 70, 71, 72. Both systems innervate many cortical and subcortical areas (Figure IB). Moreover, these systems modulate ongoing neural activity, rather than sending their own excitatory or inhibitory signals 29, 70, 73.

Although catecholaminergic systems have many similarities, they serve different functions: DA has strong projections to prefrontal and striatal areas and has mainly been associated with learning and reward-related information processing [40]. By contrast, NA mainly innervates prefrontal areas and, to a lesser extent, striatal areas [73]. It also subserves a general focussing on relevant information, irrespective of the cognitive domain [74]. However, clearer distinctions are yet to be drawn that might eventually help to diagnose impairments of either system.

## Box 2 Mechanisms of Psychiatric Disorders: From Behaviour to Neurons and Back

Psychiatric disorders are classically diagnosed based on symptom reports and clinical observations. These clinical features are rarely diagnostic of specific underlying pathological mechanisms. Here, we propose a multilevel approach to understand psychiatric disorders and their neural underpinnings. To generate hypotheses about malfunctioning brain systems, a fine-grained dissection of a patient's behaviour is important. Once consistent behavioural signatures have been found (e.g., increased response variability), we have to bridge the gap between behaviour and the neural processes that give rise to this behaviour. At the most abstract level, we formulate the key computational issue, that is, establish what problem the brain tries to solve (e.g., an optimal balance between exploiting a good foraging ground and exploring new grounds). Here, we try to answer this question from a normative perspective. Subsequently, we have to formulate how the problem is solved. At this ‘algorithmic level’, reinforcement learning has been shown to be useful [65]. Models should fulfil several requirements: (i) a good match of model predictions with the actual behaviour of an agent; (ii) model must outperform other (more simple and more complex) models in terms of model evidence; (iii) the model should have high biological plausibility (e.g., phasic DA studies lend support to RPE reinforcement learning models). Model and parameter comparison in health versus disease can then elucidate processes that underpin impairments (e.g., decision temperature parameter driving variability in ADHD; [16]). Model predictions from the algorithmic level can be used to inform data such as neuroimaging, which seeks to identify neural correlates and dynamics. By using model-derived predictions (e.g., RPEs), we can look for regions (e.g., medial prefrontal cortex) whose activity to model on the level below, thus connecting algorithmic with implementation levels. At the latter level, we can then simulate complex dynamics of neuronal systems to understand impairments. Here, we can test how problematic catecholamine systems can affect behaviour and neural activity. Thus, we can formulate new theories about neural mechanisms and potential subgroups, such as low striatal DA versus decreased frontal NA subgroups in ADHD.

Using such multilevel approaches in computational psychiatry 75, 76 helps link several levels of symptom analysis (behaviour, algorithmic, and neuronal). By finding new diagnostic subgroups, we can in principle refine therapies, based on more specific predictions about the efficacy of medication (e.g., stimulant versus nonstimulant medication) or of therapies engaging specific learning mechanisms (cognitive-behavioral therapy, neurofeedback) (Figure I).

## Box 3 How Neural Gain Affects Action Selection: The Algorithmic Level

Mathematical accounts of decision making and learning allow underlying mechanisms to be formalised in precise terms. Such formulations were first introduced during the early 20th century by Hull, Thorndike, and others, and have experienced a renaissance in recent years. Models based on reinforcement learning (RL) theory [65] have proved to be particularly useful to describe neural processes, such as phasic DA [40].

Reinforcement learning models often invoke two complementary modules: a valuation module that describes how values are learned or inferred from environmental cues, and a second module that describes an action selection process that explains how an agent selects between multiple choice options. It does this by taking the observation into account that humans and animals do not always choose the best option exploitatively, but select the option with a frequency proportional to its value (Herrnstein's matching law [77]). This is usually formulated as a softmax decision function (Equation I):

[I] $$p\left({\text{a}}_{i}\right)=\frac{e^{Q\left(a_{i}\right)/\tau }}{\sum_{k=1}^{N}e^{Q\left(a_{k}\right)/\tau }}\text{,}$$

where the probability of choosing option a_*i*_ is relative to the value of the alternative options $\left[Q\left(a_{1\ldots N}\right)\right]$. Importantly, the decision arbitration is modulated by a decision temperature parameter *τ*. This parameter moderates how deterministically the selection process follows the goodness of the choice options. In other words, the temperature *τ* dictates whether an agent strictly exploits the best option or whether it shows a more variable behaviour that allows selection of options with lower values. A low temperature parameter *τ* (Figure IA, orange) determines a high exploitative behaviour, whereas a high temperature parameter *τ* stands for an exploratory, variable behaviour (Figure IA, blue).

The neural implementation of a decision temperature *τ* (or its inverse formulation: precision) has only recently started to be studied. In decision-making and planning, *τ* is proposed to be encoded by DA 78, 79. More recent accounts of noradrenergic neural gain also render a likely modulator of a decision temperature 29, 74, 80. This is reasonable because high neural gain more strongly suppresses low-valued options and boosts high-valued options, rendering action selection more deterministic, whereas low neural gain dissociates less strongly between these options and facilitates selection of nonoptimal options.

Here, we illustrate how an increased decision temperature can mimic ADHD variability in the CPT (Figure IB, *cf* supplemental information online), where subjects have to respond when an A-X-sequence appears and an increased temperature causes ADHD-like error patterns (Figure IC).

## Box 4 How Neural Gain Changes Stability in Corticostriatal Loops

The basal ganglia are highly organised neural nuclei characterised by parallel processing. In mammals, several partially segregated corticostriatal loops have been described (Figure IA), where, for example, the cortical motor area and frontal area provide differentiated input to separate parts of the basal ganglia and receive in turn their specific processed output, via the thalamus [57].

The dynamics of these circuits are often characterised as attractor states, where the strength of the attractors scale with the strength of the feedback loop (*cf* supplemental information online). Dopaminergic drive modulates this feedback, altering the quality and strength of information conveyed from the cortex, via the striatum, through the internal pathways of the basal ganglia.

Under low DA drive, neural activity and signal differentiation in a direct and indirect pathway are comparable in strength, resulting in signal interference at the level of the output nuclei of the basal ganglia, due to the opposing information received from the two pathways (Figure IB, middle panel). This interference weakens the gain of the re-entrant system, altering the signal originally present in the cortex to the point of almost cancelling any differentiation among stimuli. Thus, weak gain is characterised by shallow attractors, where noise can easily bias activity of the network, triggering new state transitions, and resulting in high behavioural variability.

Conversely, high dopamine drive results in strong signal differentiation in the direct pathway (due to the amplification effect of strongly active D1 receptors), and weak signal differentiation in the indirect pathway (due to the compression effect of strongly active D2). Activity in the direct pathway is coherent with the gain of the loop, so that high differentiation in the direct pathway nuclei sums up with the initial differentiation present in the cortex, eventually suppressing noise and competing signals, causing behavioural stability (Figure IB, right panel).

We propose that ADHD is characterized by signal loss due to low gain. Unable to differentiate correctly among competing stimuli, selection of goals and attentional targets becomes unstable, increasing errors of both commission and omission as well as RT variability (Figure IB, bottom). Importantly, low gain in any of these loops is not necessarily associated with low DA release, and can also be caused by reduced concentration of either D1 or D2 receptors in the striatum. Pharmacological increase of the DA drive can restore the balance between signals represented in the two pathways, reducing interference and stabilising the system. Moreover, impairments of other catecholamines, such as NA, may also elicit similar effects on neural gain and behaviour [23]. Thus, it would be important to further refine the precise mechanisms in how these impairments might be dissociable on a neural or behavioural level.

## Glossary

Attention-deficit hyperactivity disorder (ADHD): a developmental psychiatric disorder characterised by inattention, hyperactivity, and/or impulsivity [5]. With a prevalence of approximately 5%, it is one of the most common psychiatric disorders during childhood [3].
Attractors, neural: when perturbed by external inputs, neural networks change the pattern of activity of their nodes (i.e., neurons). Recurrently connected neural networks exhibit nonlinear associations between inputs and patterns of activity, exhibiting state transitions towards either stable patterns (e.g., point attractors) or dynamic or complex patterns (e.g., chaotic attractors). Attractors share the common feature that different inputs converge towards the same stable or dynamic pattern and this final pattern tends to resist further input perturbation (see the supplemental information online).
Catecholaminergic system: neurotransmitter systems involving dopamine (DA) and noradrenaline (NA). The catecholaminergic nuclei are located in the midbrain (DA: ventral tegmental area and substantia nigra; NA: locus coeruleus) and project to large parts of the brain (Box 1, main text). Catecholamines are thought to modulate ongoing neural activity by modulating signal gain at the synapse.
Continuous performance task (CPT): behavioural task to test sustained attention and executive functions [10]. Participants see a sequence of random letters and have to respond when the letter combination ‘A’-‘X’ appears in sequence. For all other stimuli and stimulus combinations, participants have to withhold a response. Performance is mainly measured by their error rates as errors of commission and omission (*cf* below).
Decision temperature *τ*: the exchange rate between how much we tempt an agent (or stimulate a model neuron) and how much they change their behaviour. Say an agent is indifferent between options A versus B (or a neuron between firing versus not firing), with *τ* = US $ 10 (or *τ* = 10 mV). Adding *Δ*V = *τ* to the value of A (or *τ* to the neural input) will shift behaviour by 23% towards preferring A (or maximal firing). There are interesting reasons for not always preferring the estimated-best option, including: (i) uncertainty about its estimated value; (ii) need to explore; (iii) choice error (aka ‘trembling hand’); and (iv) ecological concerns, such as resource conservation and equity of distribution between agents. *τ* is called ‘decision temperature’ because the formula in Box 1 (main text) is a rewriting of Boltzmann's law, whereby a bigger energy gap (*cf* stimulus or reward) is required to persuade a high-temperature physical system to stay in its most likely state (*cf* preferred output or action).
Delay gratification and intertemporal choice: tasks that examine what is thought to be behavioural impulsivity. Participants have to decide between smaller rewards, which are more proximate in time, and bigger rewards, which are further away in the future. These tasks capture how much a person is impatient and devalues benefits the might arise in the future. Usually, discounting behaviour is described as a hyperbolic function with a discounting parameter k and a decision function as described in Box 3 (main text).
Error of commission: erroneous response by accidentally responding in a phase where one was to withhold one's answer. In the CPT, responses are rated as errors of commission if a response is given that does not follow an A-X letter sequence.
Error of omission: erroneous response by withholding to response to a target stimulus. In the CPT, an error of omission is counted if a participant fails to respond to an A-X letter sequence.
Marr's three level of analysis: David Marr described in his highly influential book *Vision*[7] that to fully understand how the brain solves a problem (e.g., vision), one has to explain it on three different levels: computational, algorithmic, and implementation (Box 2, main text). The computational level asks about the theoretical background; that is, about the goal of a certain computation (e.g., why do we see?). The algorithmic level asks about the mathematical implementations, so how can information be processed to solve the computational problem (e.g., recognising edges of objects). The implementation level then analyses how this is solved on a neuronal level (e.g., by orientation-specific neuronal columns).
Positron emission tomography (PET): invasive neuroimaging technique, mainly used to quantify specific receptor densities or availabilities. Due to the invasiveness and the exposure to radioactive tracers, PET is not used with children with ADHD.
Reward prediction error (RPE): hypothetical error signals originally derived from reinforcement learning theory [65]. RPEs describe the discrepancy between expected and received outcomes (or rewards) and drive learning about the value of stimuli and/or behaviours. Since the discovery that phasic DA signals in the ventral tegmental area closely reflect model-derived RPE signals [40], a huge body of literature has shown that such dopaminergically driven RPE signals are processed in multiple areas of the brain, such as the ventral striatum or the medial prefrontal lobe 66, 67, 68, 69.
