# Supplementary material for: Computational Psychiatry of ADHD: Neural Gain Impairments across Marrian Levels of Analysis
Source: Trends Neurosci. 2016 Feb;39(2):63–73. doi: 10.1016/j.tins.2015.12.009 (PMC4746317; doi:10.1016/j.tins.2015.12.009)
Supplement: Supplementary file 1 [file mmc1.pdf]

Computational psychiatry of ADHD: Neural gain impairments across Marrian levels of analysis

Supplementary material

Tobias U. Hauser, Vincenzo G. Fiore, Michael Moutoussis & Raymond J. Dolan

- How gain affects attractor state variability – a simplified graphical example
- Reinforcement Learning of CPT: illustration details
- Cortico-striatal loop and neural gain: illustration details

How neural gain drives stability – graphical depiction of a simplistic neural mass model

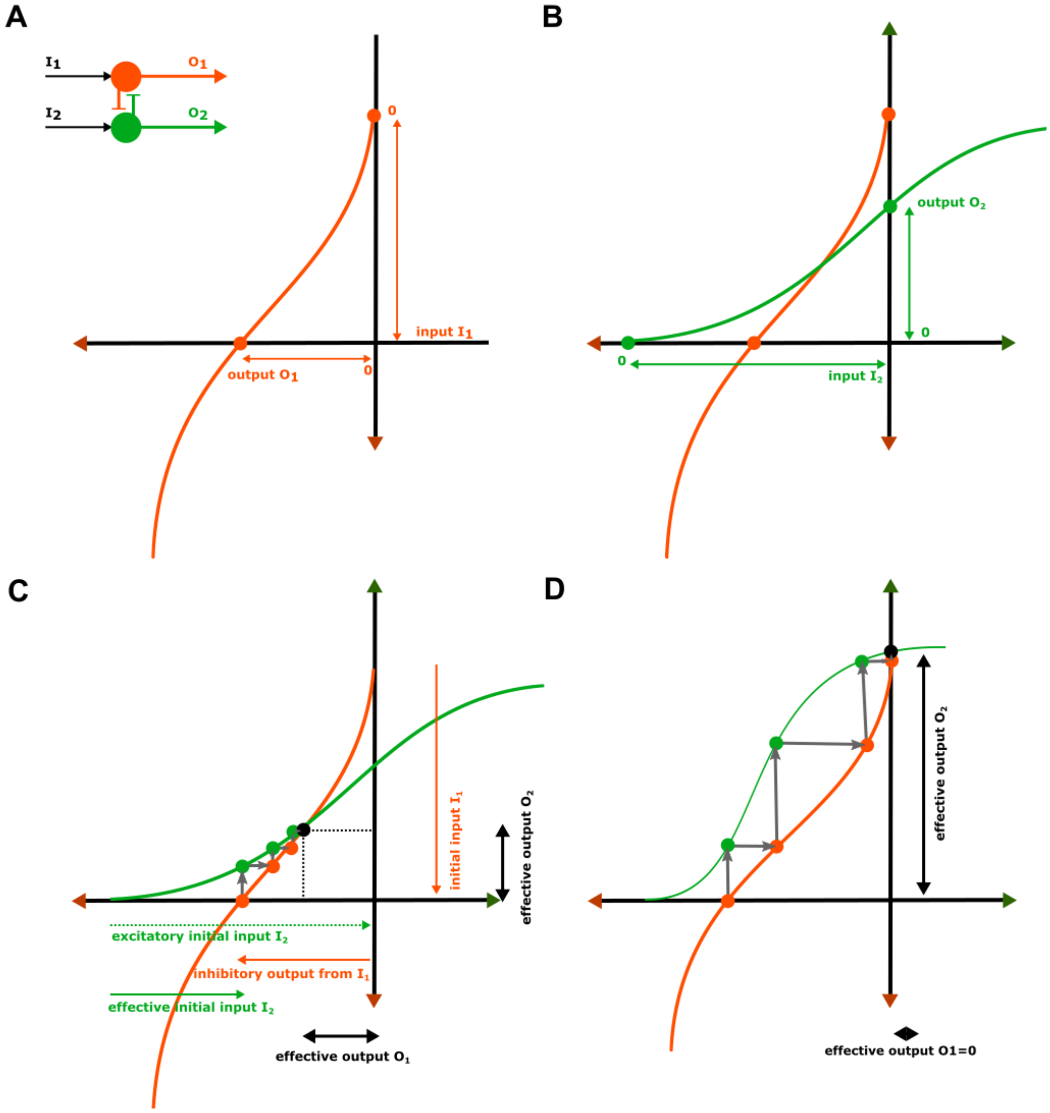

On the implementation level (Box 4), we used a complex cortico-striatal loops model to demonstrate the effects of neural gain on behavioural variability. To demonstrate how this holds true as a general principle for neural models, we demonstrate it here graphically under highly simplified assumptions. We construct a network consisting of 2 neural masses (A, top left corner). These two masses (depicted in orange and green) each receive one input (I_1, I_2), and producing outputs O_1 and O_2. These neural masses may represent two competing choice options and thus inhibit each other via mutual inhibition. As input-output functions, we use again sigmoidal function as described in Box 1. To be able to graphically depict their interaction, however, we plot this sigmoidal function rather unconventionally by rotating it (A). For neural mass 1, input into the system increases by going downwards the y-axis, where the bold orange point indicates absent input. Output of the system increases by going leftward on the x-axis. The second neural mass (green) is depicted as usual (B) with input starting from 0 at the green dot moving rightwards, and the output is depicted on the y-axis going upwards. Based on the mutual inhibition of these systems, however, the input to a system is the sum of the input and the inhibitory output from the other system. Here, we depict the evolution of the neural dynamics as an iterative process between the masses (C & D). In Figure C, we see how these systems evolve under low gain. Let's assume that neural mass 1 fires first by receiving input I_1, producing output O_1 (orange dot on x-axis). Then neural mass 2 processes its inputs. The net effective input to the system, however, is the difference between the input I_2 and the output O_1. The effective input thus is not anymore where the y-axis is depicted (as in Fig. B), but rather where the orange dot on the x-axis is placed. Using the sigmoidal function, we can easily see the output this produces, depicted as the green point on the green sigmoidal. This then again, is the effective input into system one, which will then again produce an output based on the net input. This will go on and on (as depicted by the grey arrows), until the system reaches an equilibrium state (black dot), where both populations are active, but balance each other. This means that none of the populations effectively suppresses the other and thus poorly dissociates between the two representations. This is resembled by a relatively shallow attractor surface in Box 1iii. In a high gain state (D), the sigmoidals are much steeper. This leads to a different competitive behaviour. As one can see, the activity steps are much bigger, ending relatively quickly in a state where neural mass 2 is highly active and mass 1 is completely suppressed (black dot). The system thus clearly dissociates the two options rendering it highly unlikely for option 1 to be selected. This is similar to the high gain surface in Box 1iii.

Algorithmic level: Reinforcement learning model illustration of Continuous Performance Task

To illustrate how a decreased decision temperature (i.e. lower gain) affects performance, we use the Continuous Performance Task (CPT) as an example. As a reference, we used the meta-analytically computed errors of omission and commission as described by Losier et al. (tables 7 & 8; [1]). To demonstrate the effect of the decision temperature parameter τ on behaviour, we used a simple softmax decision function (Box 3, main text) that selected between differently valued options. The action value for non-target letters (incl. 'A') was set to -.13 and for target letter sequence 'A-X' was set to .8. These settings were kept constant for both groups.

We used the (probably) most common settings for the CPT, which consist of 600 trials, 10% 'A' letters (cues) and a 50% probability of a target letter 'X' appearing after seeing an 'A'. We then ran 100 simulated agents playing the task with either a high decision temperature $\tau = .48$ (low gain) or a low decision temperature $\tau = .30$ (high gain). Results of the simulation (w.r.t. errors of commission and omission) are depicted in Box 3 as mean (errorbar: ±S.E.M.) across the agents.

Implementation level: Cortico-striatal loop models simulating effects of DA on behavioural variability

A detailed description of the model we are describing in this section and which is used for the illustrations in the main text is described in in [2–5]. To solve the task, the neural model relies on the simulation of mean-field activity in two striato-thalamo-cortical loops, interconnected at the cortical level. Each loop replicates the same intrinsic structure enabling parallel processing of the respective inputs. Semi-localistic representations in the cortex are propagated towards the input nuclei of the basal ganglia (striatum and subthalamic nucleus), maintaining the spatial representation throughout basal ganglia and thalamus due to their organisation in separate channels [6]. Information processing in the basal ganglia is enabled via three major pathways: direct pathway that is mediated via dopaminergic D1 receptors; indirect pathway, mediated by D2 receptors; and hyperdirect pathway via subthalamic nucleus. These pathways convey their respective processed inputs towards the globus pallidus pars interna (GPi, primarily for sensorimotor and associative cortico-thalamo-striatal loops) or the substantia nigra pars reticulata (SNr, for the frontal-limbic loop), output nuclei of the basal ganglia. Direct and indirect pathways both originate in the striatum, where the medium spiny neurons are enriched

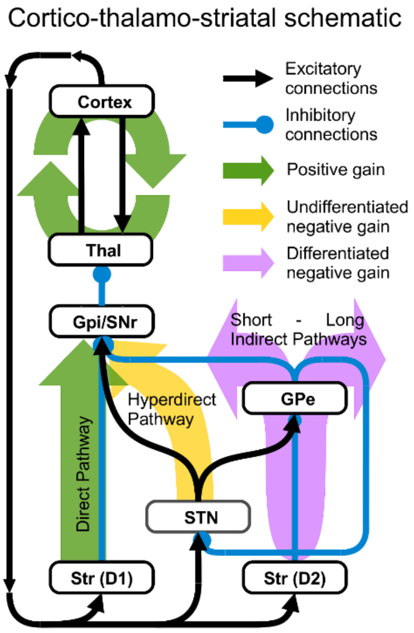

by either excitatory D1 receptors (direct pathway), or inhibitory D2 receptors (indirect pathway). Signals that originate from striatum are somatotopically organised, so that they preserve their information encoded in the cortical input concerning, for instance, salience. Conversely, the hyperdirect pathway, which is interconnected with the indirect path to form a homeostatic circuit, conveys undifferentiated tonic activity, via the sub-thalamic nucleus, to GP and SNr.

The activity of three channels is simulated in the frontal-limbic loop to represent perception and maintenance of letters A, X and third dummy letter (representing all other letters). In the sensorimotor loop, the activity in two channels simulates the competition between two motor representations: the action of pushing the space bar and a dummy action. As described before [2,3,5], leaky integrator is used to simulate the average activity of an entire pool of neurons.

A three dimensional vector is used as external input for both cortico-thalamo-striatal loops: stimuli for salient letters (A and X) are represented as noisy values in a range of values of 0.6±0.1, lasting 750ms. Non-salient letters are all represented as noisy stimuli in a range of 0.3±0.1, for the same duration. Interval between stimuli is fixed to 1500ms and it is characterised by noise in the range 0.1±0.1. The input reaches the frontal-limbic loop via parallel projections, so that each input activates only one channel. Conversely, all inputs projects towards a single action-channel in the sensorimotor loop ("push" action). The task only rewards one action selection, when performed under the correct conditions, so that this choice about input connectivity simulates the status of the system after the learning process is complete. The same reasoning applies for cortico-cortical connectivity, where the second layer of the cortex in the frontal-limbic loop is connected with the unit in the inner cortical layer of the sensorimotor loop representing the action "push". Learning processes leading to this type of connectivity have been simulated and discussed in previous work [2,5]. In a normal gain condition, letters A and X trigger a winnerless competition [7] in the frontal loop due to the high gain, so that the presence of the salient stimulus is maintained in a memory-like phenomenon until a new salient stimulus is presented. When letter X is perceived after letter A, both representations are combined, triggering the appropriate action. Conversely, an ADHD-like condition (low gain) is characterized by interference in the frontal loop, which cancels the differences in value or salience among perceived inputs. The resulting selections and maintenance are more stochastic and strongly influenced by neural noise, causing the increase in errors of commission and omission. Finally, an increase in striatal DA offsets the interference effect by unbalancing the information represented in the two pathways in favour of the direct pathway. The frontal system can therefore re-establish its routine of selection and correct maintenance, resulting in a decrease in motor errors.

References

1 Losier, B.J. *et al.* (1996) Error patterns on the continuous performance test in non-medicated and medicated samples of children with and without ADHD: a meta-analytic review. *J. Child Psychol. Psychiatry* 37, 971–987

2 Fiore, V.G. *et al.* (2014) Keep focussing: striatal dopamine multiple functions resolved in a single mechanism tested in a simulated humanoid robot. *Front. Psychol.* 5, 124

3 Fiore, V.G. *et al.* (in revision) Changing pattern in the basal ganglia: motor switching under reduced dopaminergic drive.

4 Fiore, V.G. *et al.* (2015) Evolutionarily conserved mechanisms for the selection and maintenance of behavioural activity. *Philos. Trans. R. Soc. Lond. B. Biol. Sci.* 370,

5 Baldassarre, G. *et al.* (2013) Intrinsically motivated action-outcome learning and goal-based action recall: a system-level bio-constrained computational model. *Neural Netw. Off. J. Int. Neural Netw. Soc.* 41, 168–187

6 Alexander, G.E. *et al.* (1986) Parallel organization of functionally segregated circuits linking basal ganglia and cortex. *Annu. Rev. Neurosci.* 9, 357–381

7 Rabinovich, M.I. *et al.* (2006) Dynamical principles in neuroscience. *Rev. Mod. Phys.* 78, 1213–1265
